# Cluster of *Mycobacterium smegmatis* mastitis cases in a dairy herd incorporating recycled manure solids bedding

**DOI:** 10.3389/fvets.2025.1704276

**Published:** 2026-01-26

**Authors:** Heli Lindeberg, Jaana Seppänen, Lilli Frondelius, Tarja Pohjanvirta, Tiina Autio

**Affiliations:** 1 Production Systems, Natural Resources Institute Finland, Maaninka, Finland; 2 Finnish Food Authority, Animal Health Diagnostic Unit, Helsinki, Finland; 3 Finnish Food Authority, Animal Health Diagnostic Unit, Kuopio, Finland

**Keywords:** bovine, intramammary infection, *Mycobacterium smegmatis*, recycled manure solids, whole genome sequencing

## Abstract

This study examined uncommon mastitis cases in a research barn incorporating recycled manure solids (RMS) as bedding. The cases occurred after barn renovation, including the conversion of the herringbone milking parlor to an automatic milking system (AMS) and the replacement of rubber mattress stalls with deep-bedded stalls maintained daily with RMS. Cows were milked at the herringbone milking parlor until the automatic milking system was available. Approximately 6 months after the implementation of AMS and deep-bedded stalls, the first two cows exhibited cow level somatic cell counts ≥ million cells/ml and palpable hardness in udder quarters manifested. However, commercial quantitative polymerase chain reaction testing, typically employed for mastitis diagnostics in Finland, did not identify pathogens in the quarter milk samples. *Mycobacterium smegmatis* was isolated through bacterial culturing. Within 9 months, five more *M. smegmatis* mastitis cases occurred in the dairy barn. Whole genome sequencing (WGS) of the isolates revealed considerable genetic diversity among strains. However, chronic infections in individual quarters caused by persistent strains were also detected. The WGS-based core-genome multilocus sequence typing approach demonstrated its efficacy as a robust tool for the molecular epidemiological exploration of bovine non-tuberculous mycobacterial mastitis. All lactating cows were tested for *Mycobacterium avium* subspecies *paratuberculosis* antibodies using an enzyme-linked immunosorbent assay test. Only the cows with *M. smegmatis* mastitis gave a positive reaction, although the causative agent of paratuberculosis was not detected in their fecal samples.

## 1 Introduction

The dairy sector is interested in alternative bedding materials that would be cost-effective, easily available in large quantities, and safe for animals and humans (1). Recycled manure solids (RMS) are used in several countries in deep bedding and on top of mattresses in lying stalls (2, 3). However, little information is available regarding the suitability of the material under Nordic farming conditions. Additionally, under Nordic farming conditions, scientific knowledge of the possible effects of RMS on cow health is currently scarce (4, 5). RMS is an organic bedding material with a high moisture content (6) and a naturally high bacterial content (7–10). RMS provides conditions that support bacterial growth to counts that have been reported to be greater with RMS than with other common bedding materials (11). Due to the nature of RMS, there is concern about the number of pathogens it contains and the risks it poses to the health of cows and farm staff. Mastitis pathogens such as coliforms, *Klebsiella* species, *Streptococcus* species and *Staphylococcus* species (12–16) as well as zoonotic pathogens such as *Cryptosporidium* spp., *Salmonella* spp., and *Listeria monocytogenes* have been isolated from RMS (16–18). In addition, *Mycobacterium avium* subspecies *paratuberculosis* (MAP), the causative agent of Johne’s disease, i.e., paratuberculosis, has been found in raw slurry (19, 20), in slurry and manure after fermented for up to 2 months (21) and in samples of unused RMS (2, 16).

Several studies have been focused on the effect of RMS bedding to udder health (13, 14, 16, 22–24). Various RMS bedding production procedures exist, of which some include steps such as maturation that can mitigate the microbial risk (16). This hampers the comparison of the studies. In addition, the majority of the comparisons have been conducted between RMS and sand, and few studies have compared RMS with other organic bedding materials. There are studies reporting either no association between the type of bedding and the occurrence of clinical or subclinical mastitis (2, 8, 25, 26) or a more than two times greater risk of developing clinical mastitis on RMS (2, 9). In the studies of Frondelius et al. (4) and Jeppsson et al. (5), the use of RMS was associated with a statistically nonsignificant increase in the incidence of clinical mastitis compared to peat or wood shavings. Fréchette et al. (1) noticed that the general incidence of clinical mastitis and especially severe clinical mastitis was not higher when using RMS bedding compared with straw bedding. However, the risk of clinical mastitis caused by *Klebsiella pneumoniae* was seven times higher in cows on RMS bedding compared to cows on straw bedding, which is in concordance with reports of veterinarians and milk producers on the use of RMS (27). *Klebsiella* mastitis cases are typically severe and can lead to the death or premature culling of the cow (28). Even though clinical mastitis is an important outcome of udder health, it is problematic as it depends on the ability of the farm to identify and record clinical mastitis cases. Beyond clinical mastitis, other important measures of udder health such as subclinical mastitis, based on somatic cells count or linear cells score, have been studied. Patel et al. (9) demonstrated poorer udder health measures with RMS compared to organic non-manure materials, reclaimed sand, or new sand bedding materials. In Canadian study, cows on RMS farms had 0.73 times the risk of acquiring subclinical mastitis when compared to straw-bedded farms (25). However, this was not statistically significant.

With the use of RMS and different types of compost substrates as a bedding material, a new group of occasional mastitis cases caused by fast-growing non-tuberculous mycobacteria (NTM) has emerged (29, 30). *Mycobacterium smegmatis* (also named *Mycolicibacterium smegmatis* (31)) has typically been considered as an opportunistic causative agent of mastitis in individual animals. It is a saprophyte in the soil and can enter the udder via the teat canal. The first references to mastitis in dairy cows caused by NTM date from the 1950s (32, 33). Since then, few reports on *M. smegmatis* mastitis have been published (34–37). Only two reports have demonstrated a possible association between *M. smegmatis* mastitis and the bedding material used. According to Ghielmetti et al. (29), compost bedded pack was potentially associated with a sudden increase in clinical mastitis cases in a herd, two of which were caused by *M. smegmatis*. Similarly, in a study by Supré et al. (30), two cows with symptoms of mastitis were diagnosed with *M. smegmatis* infection, and RMS was presented as a possible source of these environmental mastitis infections.

In this paper, we describe a cluster of *M. smegmatis* mastitis cases in a research dairy herd of Natural Resources Institute Finland (Luke). The isolates were characterized by whole genome sequencing (WGS). To our knowledge, this is the first study using core-genome multilocus sequence typing (cgMLST) to characterize NTM isolates originating from bovine mastitis.

## 2 Method

### 2.1 Housing conditions

The data were collected in the research barn of Luke (Maaninka, Finland, 63 °10 ‘N, 27 °18′E), which is a free-stall curtain-wall barn with automatically scraped (Lely Discovery 90SW, Lely, The Netherlands) slatted passageways. In 2021–2022, the barn underwent a major renovation in which the 2 × 8 herringbone milking parlor was converted to an automatic milking system (AMS; DeLaval VMS V300, Sweden) and the herd size was reduced from 120 to 60 dairy cows, compatible with one AMS unit. In the dairy cow compartments, the stalls with 4-cm-thick mattresses having a rubber covering (Soft Bed 4GS, Huber Technik GmbH and Co. KG, Germany) bedded thrice weekly with peat or RMS (4) were changed to deep-bedded stalls (Ergo XL, Pellon Group Oy, Finland) bedded daily with RMS. Additionally, close-up and fresh cows were provided with a deep-bedded pack area (sand + straw). All cows had free access to total mixed ration and water. Initially, manure was separated with a screw separator (Bauer Separator S 655, Bauer GmbH, Austria) with a sieve size of 0.5 mm. The dry matter (DM) content of unused RMS was analyzed by drying the samples (100 g) in oven in +100 °C for 24 h (4). The DM content varied between 24 and 28%. During spring 2023, the original separator was replaced with an EYS SP800HD-W screw-press separator (EYS Endüstri Makina San ve Tic. A.Ş., Turkey). After adjustments, which included replacement of a sieve with a smaller opening size of the mesh, feeding a larger amount of raw slurry, reducing the flow and pressure of the feed pump and reducing the number of counterweights (approximately 8 kg each) to two per side, the DM content of RMS increased to approximately 34–38% in August 2023. Raw nondigested slurry was separated into raw solid fraction which was immediately or within the same working day used as bedding without composting. In summer 2022, all lactating cows were enrolled in a grazing experiment in which the fixed daily pasture time was four or six hours and all cows were simultaneously on pasture. Body condition score (BCS) was recorded using DeLaval BCS camera (DeLaval, Sweden) between 16th March 2023 and 31st December 2023; *n* = 10,311 daily BCS recordings of 32–42 cows (containing 26–42 Holstein (*n* = 8,527 recordings) and 6–8 Nordic Red cows (*n* = 1,784 recordings)).

### 2.2 Herd characteristics

The data were collected in 2022 and 2023. The average herd characteristics from the years 2021–2023 are presented in Table 1 as they are reported in official milk recording (ProAgria, Finland), which is collected according to the International Committee for Animal recording guidelines (38).

**Table 1:** Average herd characteristics of the study herd in 2021–2023, presenting the milk yield, somatic cell count, parity, breed distribution, calving interval, and culling rate as reported in official milk recording.

Herd characteristic	2021	2022	2023
Number of cows	63.1	57.5	65.9
Total milk yield, kg/cow	9,573	10,088	9,652
Sold milk, kg/cow	8,607	8,856	8,663
Milk fat, % (Holstein/Nordic red)	5.02 (4.93/5.21)	4.36 (4.26/4.63)	4.43 (4.38/4.66)
Milk protein, % (Holstein/Nordic red)	3.59 (3.54/3.70)	3.54 (3.49/3.68)	3.55 (3.52/3.69)
Cow level somatic cell count, x1,000/mL	156	203	191
Body condition score (Holstein/Nordic red)	NA	3.19 (3.12/3.50)	NA
Parity	2.46	2.63	2.64
Holstein/Nordic red, %	69.4/30.6	71.5/28.5	78.1/21.9
Calving interval in days	391	392	414
Culling, %	60^1^	23	21

^1^The herd size was reduced from 120 to 60 dairy cows, compatible with one AMS unit.

NA, Not available.

### 2.3 Udder health management

For each bulk tank milk collection, the milk composition, somatic cell count (SCC), and total number of bacteria are analyzed by the regional laboratory of Valio Ltd. in Seinäjoki. Monthly milk samples from individual lactating cows are collected to determine the composition of the milk (SCC, number of bacteria, fat, protein, urea, and lactose). The DeLaval Delpro™ FarmManager monitors the SCC of individual cows and reports the cows with possible mastitis; the DeLaval OCC online cell counter provides 1–3 cow level SCC readings daily, depending on how many times the cow is milked.

All treatments of subclinical (cow level SCC > 200,000 cells/mL), clinical mastitis and selective dry cow therapy are based on pathogen analysis of quarter milk samples. The California Mastitis Test (score 1–5) is performed on all suspected mastitis cases. The treatments are prescribed according to the guidelines (39) of good antibiotic practices followed in the country and considering whether the pathogen is a penicillin-sensitive or a penicillin-resistant bacterial strain. Pathogen-based therapy is applied for both subclinical and clinical mastitis and drying off. Treatment protocols of subclinical and clinical mastitis: *Streptococcus* spp., and *Staphylococcus* spp. mastitis: A 4-day twice a day intramammary treatment. Cases infected by penicillin-sensitive bacteria are treated with benzylpenicillin procaine (Carepen vet 600 mg, Vetcare, Finland) and cases infected by penicillin-resistant strains are treated with ampicillin/cloxacillin sodium (Ampiclox vet 75/200 mg, Zoetis Finland). *E. coli* and *Klebsiella* spp. mastitis: 3 to 5-day parenteral treatment with enrofloxacin (Fenoflox vet 100 mg/mL Vet Medic Animal Health, Finland or Baytril vet 100 mg/mL Elanco, Denmark). Treatment protocols of drying off: All cows are treated with internal teat sealants containing bismuth subnitrate (either Noroseal 2,6 g, Vet Medic Animal Health, Finland or Ubroseal vet 2,6 g, Vetcare, Finland). With no bacterial findings at drying off, a cow is treated with internal teat sealants only. Based on bacteriologic results one treatment with either penetamate hydroiodide 100 mg/benethamine penicillin 280 mg/framycetin sulfate 100 mg; (Umpimycin vet, Vetcare, Finland) for quarters infected by penicillin-sensitive strain or cloxacillin (Orbenin retard vet 500 mg, Zoetis Finland) for quarters infected by penicillin-resistant strain is used.

Treatment data of cows was obtained from the Centralized Health Care Register for Finnish Cattle Herds (NASEVA) and cow level SCC data, BCS data, milking data (drying off the *M. smegmatis* quarters) were obtained from the DeLaval Delpro™ FarmManager. The key results are shown in Table 2.

**Table 2:** Characteristics of *Mycobacterium smegmatis* mastitis cows.

Cow ID	Cow 1	Cow 2	Cow 3	Cow 4	Cow 5	Cow 6	Cow 7
Breed	NR	NR	NR	NR	NR	NR	H
Number of calvings	5	3	4	4	4	4	2
BCS (at dry off/a week after calving)	3.88/3.72	3.48/3.72	3.46/3.64	3.77/3.85	ND/3.69	3.70/3.72	3.42/3.40
305 d milk yield (kg)	8993	9522	11347	10548	7682	11279	7530
Milk fat % in 2022–2023^1^	4.56	5.01	4.86	4.50	4.78	3.91	4.79
Treated quarters during lactation^2^	No treatment	LF, RF	LF, LH, RH	LF, RF, RH	LH, RH	RH	No treatment
*M. smegmatis* isolation:							
Date (1st isolation)	22.10.2022	22.10.2022	5.12.2022	5.12.2022	3.3.2023	5.5.2023	3.7.2023
Days in milk (DIM)	42	38	48	37	14	117	7
Highest SCC (x1,000/mL)	1405	1020	1602	2660	1185	862	1137
Milk yield (kg)	34.3	39.7	33.5	47.8	42.9	41.6	32.4
Quarter(s) and CMT score	LH5	LF5	LF5	LF5	RF3	RH5	LH5
		RF5	RH5	RF5			
DIM at culling	188^3^	279	84	100	123	23^4^	265^5^
MAP antibody ELISA^6^	pos (S/P % 49)	NA	NA	NA	NA	pos (S/P % 74)	pos (S/P % 86)
MAP qPCR	neg	NA	NA	NA	NA	neg	neg

*M. smegmatis, Mycobacterium smegmatis;* NR, Nordic Red; H, Holstein; LF, left forequarter; LH, left hindquarter; RF, right forequarter; RH, right hindquarter; CMT, California Mastitis Test (score 1–5); S/P %, sample/positive % (S/P % ≤ 30% negative; S/P % > 30% positive); MAP, *Mycobacterium avium* subspecies *paratuberculosis*; ELISA, enzyme-linked immunosorbent assay; ND, Not detected at dry off; NA, Not available, cow was culled before sample collection; BCS, body condition score; DIM, days in milk; SCC, somatic cell count (cow level); qPCR, quantitative polymerase chain reaction. ^1^Average milk fat % for 365 days (2022: Cows 1–4; 2023: Cows 5–7). ^2^a 4-day twice a day intramammary treatment before *M. smegmatis* isolation using either benzylpenicillin procaine or ampicillin and cloxacillin sodium salt. ^3^DIM at culling after 6th calving, isolation during the 5th lactation. ^4^DIM at culling after 6th calving, isolation during the 4th lactation. ^5^DIM at culling after 3rd calving, isolation during the 2nd lactation. ^6^Samples collected in July 2023.

### 2.4 Sampling of mastitis

Quarter milk samples were routinely and aseptically taken by research barn staff from all subclinical and clinical mastitis cases. Sample tubes containing the preservative bronopol were used, and the samples were sent to the Valio regional laboratory for analysis of pathogens using the quantitative polymerase chain reaction (qPCR) method (Thermo Scientific PathoProof Mastitis Complete-16 assay, Thermo Fisher Scientific Ltd.). Targeted species in this method are *Corynebacterium bovis*, *Enterococcus faecalis/faecium*, *Escherichia coli*, *Klebsiella oxytoca/pneumoniae*, *Serratia marcescens*, *Staphylococcus aureus*, *Staphylococcus* spp., *Streptococcus agalactiae*, *Streptococcus dysgalactiae*, *Streptococcus uberis*, *Peptoniphilus indolicus/Trueperella*, *Mycoplasma bovis*, *Mycoplasma* sp., yeasts, and *Prototheca* spp.

In October 2022, one clinical mastitis case yielded no pathogen findings in mastitis qPCR and the quarter was resampled for bacterial cultivation, resulting in the isolation of *M. smegmatis*. Subsequently, duplicate quarter milk samples were taken in every mastitis case. One tube with preservative was analyzed with qPCR and the other sample without preservative was stored frozen (−20 °C) immediately after sampling. Based on qPCR results, the milk samples with no findings were brought frozen to the laboratory of the Finnish Food Authority for bacterial culture.

### 2.5 Bacterial cultivation and identification

A total of 10 μL of milk underwent conventional aerobic cultivation [Tryptic soy agar with 5% defibrinated sheep blood (TSA, CASO agar, Merck, Darmstadt, Germany)] at 37 °C for 120 h. Growth of smooth to mycoid, small white colonies was observed after 3 d of incubation. The isolates from pure growth were identified using matrix-assisted laser desorption/ionization time-of-flight mass spectrometry (MALDI-TOF MS) analysis. The analysis was performed by direct spotting according to the manufacturer’s instructions with a MALDI Biotyper® instrument (Bruker Daltonics GmBG, Germany) using MBT Compass software and by using Bruker MBT Compass Library database revision 2021. A score of over 2.0, indicating high confidence in species-level identification, was applied (40).

### 2.6 *Mycobacterium avium* subsp. *paratuberculosis* (MAP) detection and antibody enzyme-linked immunosorbent assay (ELISA)

A total of 51 composite milk samples were collected for antibody analysis from all lactating cows in July 2023. The samples were analyzed with a commercial ELISA test (ID Screen® Paratuberculosis Indirect Screening test, IDVet, France) according to the manufacturer’s instructions. From all the ELISA-positive cows (*n* = 3), fecal samples were collected and analyzed for the presence of MAP by qPCR (21, 41). Briefly, genomic DNA was extracted using the QIAamp PowerFecal Pro DNA Kit (Qiagen, Hilden, Germany) following the manufacturer’s instructions. Real-time PCR assays targeting the *IS900* and *F57* genes (21, 41) were performed on a CFX96 real-time PCR detection system (Bio-Rad Laboratories, CA). Plasmid pUC18 was included as an internal amplification control, as described by Fricker et al. (42).

### 2.7 DNA extraction, WGS, and characterization of the isolates using cgMLST

A total of 10 isolates from six cows were characterized. After growing isolates for 72 h on blood agar as described above, DNA was extracted using the DNeasy Blood & Tissue kit (Qiagen) in a QIAcube classic instrument (Qiagen) according to the manufacturer’s enzymatic lysis protocol for Gram-positive bacteria. The integrity of the DNA was controlled with agarose gel electrophoresis and the concentration was quantified using the Qubit dsDNA BR Assay Kit (Thermo Fisher Scientific, Waltham, USA). The library for WGS was prepared using the Illumina DNA Prep kit (Illumina, San Diego, CA, USA) and the DNA was sequenced using Illumina V2 2 × 250 bp chemistry and a MiSeq benchtop sequencer (Illumina). Adapters were removed using Local Run Manager software (Illumina). FASTQ quality checking was performed using FastQC (FastQC version 0.11.7, quality control for raw sequence data; GPL v3, https://www.bioinformatics.babraham.ac.uk/projects/fastqc).

Species identification was confirmed by ribosomal multilocus sequence typing (43) and the average nucleotide identity was calculated using the OrthoANIu algorithm [https://www.ezbiocloud.net/tools/ani; (44)]. A threshold of 96% identity was used for species delineation (45).

Short reads were trimmed to Q ≥ 30 and *de novo* assembled into contigs using the Velvet algorithm in Ridom SeqSphere+ software v 7.1.0 (Ridom, Münster, Germany). The isolates were genotyped using cgMLST analysis, with cgMLST schema targets covering 70.1% of the reference genome. The scheme for the *ad hoc* cgMLST was defined using a target definer tool within Ridom SeqSphere+ software to identify 4956 target loci from the reference strain NZ_CP054795.1 (*M. smegmatis* FDAARGOS_679) and eight complete query genomes obtained from GenBank (Supplementary material 1). The genome sequences were subjected to local ad hoc cgMLST analysis using Ridom SeqSphere+ software. The 4,956 cgMLST allele-called targets of the 10 isolates were extracted and compared with each other. An average coverage of >40 in combination with >95% of good cgMLST targets is considered acceptable sequence quality. The minimum spanning tree within Ridom SeqSphere+ software was used to visualize allelic differences. The minimum spanning tree was created with the setting of pairwise ignoring missing values. The cgMLST results were combined with the epidemiological information. The sequence data are available in the European Nucleotide Archive (ENA) database under study project PRJEB83779.

## 3 Results

### 3.1 The course of *Mycobacterium smegmatis* mastitis in the herd

In 2021–2022 the research barn underwent a major renovation, including the conversion of the herringbone milking parlor to an automatic milking system (AMS) and the replacement of rubber mattress stalls with deep-bedded stalls maintained daily with RMS. The dry matter content of RMS varied between 24 and 28%. Cows were milked at the herringbone milking parlor until the automatic milking system was available. Approximately 6 months after the implementation of AMS and deep-bedded stalls, the first two cows exhibited cow level milk somatic cell counts ≥ million cells/ml and palpable hardness in udder quarters manifested. However, commercial qPCR testing did not identify pathogens in the milk and *M. smegmatis* was isolated through bacterial culturing. Within 9 months, five more *M. smegmatis* mastitis cases occurred in the dairy barn. At the time of the permanent increase in the milk SCC above 200,000 cells/mL, the cows were housed either in the compartment for lactating cows, where they had access to deep beds (*n* = 5 cows), or in a bedded pack area (*n* = 2 cows).

A total of seven cows had *M. smegmatis* mastitis in 10 quarters. These cows had variable udder health status in the previous lactation period as well as other characteristics including DIM, parity, and breed (Tables 2, 3). The average BCS of Holstein and Nordic Red cows in the herd was 3.12 and 3.50, respectively (Table 1). Compared to herd BCS averages, all the infected cows had higher BCS 1 week after calving (average 3.68) and at drying off (average 3.62, days in milk (DIM) range 300–362 days, Table 2).

**Table 3:** Characteristics of the quarter milk samples with no findings in the quantitative polymerase chain reaction (qPCR) test but containing *Mycobacterium smegmatis* in bacterial culture.

**Quarter milk samples (*n*)**	13
Nordic Red cows (*n*)	12
Holstein cows (*n*)	1
California Mastitis Test (mean ± SD, score 1–5)	4.6 ± 0.7 (*n* = 12)
Cow level SCC^1^ (x10^6^/mL) (mean ± SD)	1,342 ± 604 (*n* = 8)
Days in milk (mean ± SD)	43 ± 36 (*n* = 7)^2^
**Cows (*n*)**	7
Nordic Red cows (*n*)	6
Holstein cows (*n*)	1
Calvings (mean ± SD)	3.6 ± 1.3

^1^SCC, somatic cell count. SCC of 5 *M. smegmatis* quarters was not registered due to malfunctioning of the OCC online cell counter. ^2^Days in milk at first isolation of *Mycobacterium smegmatis*.

An increase in the milk SCC to over one million cells/mL (Table 2) was observed in the affected cows, but none of them had fever or other general symptoms. SCC was not available for 5 *M. smegmatis* quarters (Table 3) as the OCC online cell counter malfunctioned, and no SCC was registered. Typically, the affected quarter was hard, its California Mastitis Test value was at the highest score of 5, and some cows had flakes in the milk. In five cows, prior to *M. smegmatis* isolation, there was a non-aureus *staphylococci* (NAS) infection, which was treated with intramammary antibiotics. Regardless of the treatment, the milk SCC remained high in these cases, and new milk samples were taken from the affected quarters for qPCR and tested without pathogen findings.

No antibiotic treatments were applied to *M. smegmatis* infected quarters, and the infected quarters were dried off, or drying off was initiated even if the cow was culled immediately after the *M. smegmatis* diagnosis. In the cows remaining in the herd, dried off *M. smegmatis* quarters were *M. smegmatis* positive also after their subsequent calvings.

All 51 lactating cows in the herd were tested for MAP antibodies using the ELISA test. Three cows diagnosed with *M. smegmatis* mastitis were still in the herd at the time of the sample collection and had S/P % values above the cut-off (Table 2). Fecal samples were taken from these cows and tested by qPCR targeting MAP, with negative results.

### 3.2 *M. smegmatis* identification and cgMLST

**Figure 1:**
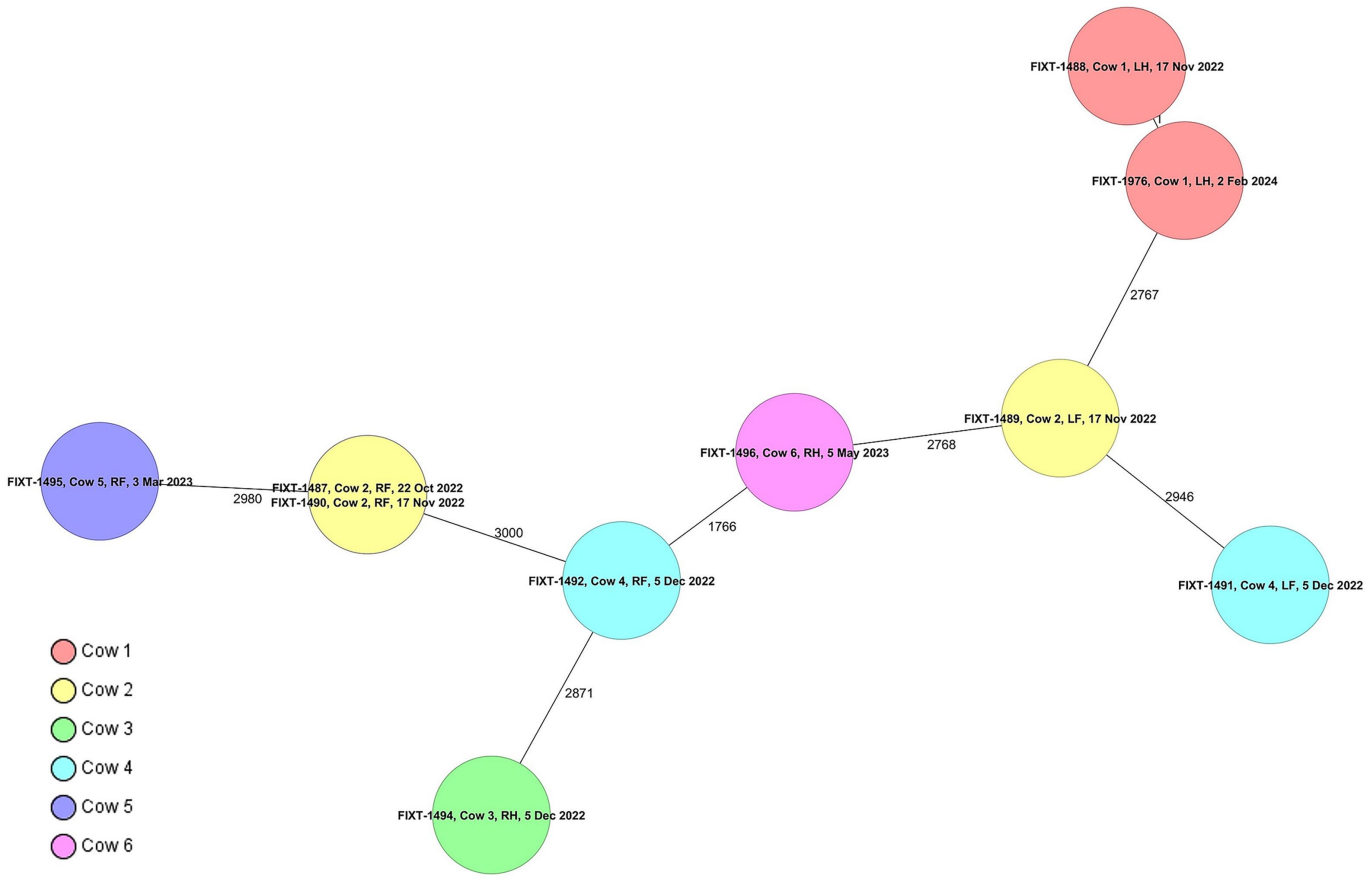
The minimum spanning tree based on core-genome multilocus sequence typing (cgMLST) allelic profiles of the isolates. Each circle represents one cgMLST profile, with the isolate ID(s), the cow ID(s), the quarter(s), and the date(s) of isolation indicated. The numbers between the circles denote the allelic difference between the profiles.

The suspected isolates were identified using MALDI-TOF, and identity scores over 2.0 for *M. smegmatis* were obtained for all isolates. Ribosomal multilocus sequence typing and average nucleotide identity analyses were used to confirm species identification. A minimum average nucleotide identity value of 98.82% was obtained, indicating correct species identification, as a threshold of 96% identity has been suggested for species delineation (43). cgMLST revealed high diversity among *M. smegmatis* strains. Allele differences between 1,766 and 3,156 alleles were observed between the majority of the strains, and all cows had a unique *M. smegmatis* cgST/profile (Figure 1). Cows 2 and 4 had an infection in two udder quarters, and these were different cgSTs. However, in two cows (cows 1 and 2), the same quarters were sampled twice, with the sampling interval being 1 month (cow 2, FIXT-1487 and FIXT-1490) and over 1 year (cow 1, FIXT-1488 and FIXT-1976). In these cases, chronic persistent infection caused by a single strain within a cow was confirmed by cgMLST.

## 4 Discussion

NTM have been described as uncommon causative agents of bovine mastitis (29, 30, 46–51). Recently, several publications have highlighted the role of *M. smegmatis* (29, 30, 48), but to our knowledge there are no studies on genomic diversity of the isolates. Here, we describe seven cases of *M. smegmatis* mastitis in a dairy herd incorporating recycled manure solids as bedding and the genetic characterization of *M. smegmatis* isolates.

In our case, *M. smegmatis* mastitis was associated with a high SCC (typically millions of cells/mL), swollen and indurated infected udder quarters, either yellowish mucous flakes in the foremilk secretions or no changes in milk, and no general symptoms. This is in accordance with previously reported cases of *M. smegmatis* as the cause of sudden, latent, and long-term treatment-resistant mastitis in cattle (29, 30, 46–49). The prevalence of the cases is most likely underestimated, as mycobacteria may easily go undiagnosed when using multiplex mastitis PCR diagnostics or be misdiagnosed, as NTM is an unusual finding as a cause of mastitis in cattle. The reports of Machado G et al. (50), Machado CS et al. (51), Ghielmetti et al. (29), and Supré et al. (30) support our perspective. There is reason to suspect mycobacteria as a possible cause of mastitis in cases where cows have had a previous unsuccessful antimicrobial treatment or a negative result in multiplex PCR for common mastitis pathogens from a quarter with a high SCC (millions of cells/mL).

WGS has recently been successfully utilized for outbreak and transmission analysis of rapidly growing NTM infections in humans (52–55). To our knowledge, this is the first study using cgMLST to characterize NTM isolates originating from bovine mastitis. In our case, the isolates displayed high diversity, having in the majority of cases 1,766–3,156 allele differences. Diricks et al. (54) similarly reported over 2000 allele differences among both *Mycobacterium abscessus* subsp. *massiliense* and subsp. *abscessus* strains originating from distinct outbreaks in humans. Their study consisted of WGS data from a total of 1991 *Mycobacterium abscessus* isolates. It appears that as NTM bacteria are ubiquitous in natural aquatic and soil environments, they have high genetic diversity, as demonstrated by cgMLST. However, low within-patient diversity revealed infection with a highly persistent strain for a decade without any allelic difference (54). We recorded a similar finding regarding within-quarter diversity in cows. In two cows (cows 1 and 2), the same quarters were sampled twice, with the sampling intervals being 1 month (cow 2) and over 1 year (cow 1), yielding highly similar sequence types (ST) with a difference of zero and one allele. However, when examining different udder quarters within a cow, a unique ST was isolated, indicating separate infections transmitted via the teat canal.

The clinical cure of *M. smegmatis* mastitis remains uncertain, and antibiotic therapy seems to usually fail (29, 36, 47, 48). In our case, no antibiotics were administered to cows with *M. smegmatis* mastitis. Four out of seven infected cows were culled due to multi-quarter chronic infections. Three cows had only one infected *M. smegmatis* quarter, and the infected quarters were dried off. After calving, the infected quarters remained *M. smegmatis* positive and were re-dried. To avoid empirical antibiotic treatment and inappropriate use of antimicrobials, performing sampling and pathogen detection at the onset of the mastitis cases is important. The identification of mastitis pathogens is often performed either by standard bacterial culture using 48 h incubation time for culture plates, or using commercial mastitis pathogen qPCR kits, which detect limited selection of pathogens. The diagnosis of *M. smegmatis* infections may thus be difficult, as it requires 3–5 days incubation time for culture plates, and it is not included as a target pathogen in commercial mastitis qPCR kits. In cases of clinical mastitis showing negative tests for the usual bacterial causative agents, NTM should be suspected as the possible uncommon causative agent.

The limitations of this study were a small sample size and observational study design. Therefore, it is not possible to clarify the cause of the infections. The bedding material has been discussed as a possible environmental source of *M. smegmatis* mastitis (29, 30). Ghielmetti et al. (29) described two cases of *M. smegmatis* mastitis in a Swiss dairy herd after compost bedded pack change. The bedding material, consisting of sawdust and woodchips mixed with organic biodegradable waste, was proposed as a possible risk factor. Environmental origin was also discussed by Supre et al. (30), who demonstrated two *M. smegmatis* mastitis in cows housed on recycled manure bedding in deep stalls in Belgian dairy herd. In our case, *M. smegmatis* mastitis cases occurred approximately 6 months after the implementation of deep-bedded stalls with RMS, which had low dry matter content. However, no supporting microbiological analyses from bedding material were performed to confirm the presence of *M. smegmatis.* As stated by Jeppsson et al. (5), the high level of total bacteria in the RMS bedding material requires attention to bedding and milking routines as well as regular monitoring of herd health.

Previously, antimicrobial intramammary treatment of the udder quarter was suspected to be the cause of mycobacterial mastitis (30, 36, 48). Supré et al. (30) described *M. smegmatis* mastitis in cows which have been treated intramammary for prolonged period. In the study of Thomson et al. (36), a total of 59% of cows treated intramammarily for subclinical streptococcal mastitis developed *M. smegmatis* mastitis 3 to 6 weeks after the treatment. Intramammary treatment was suspected to contribute to the onset of mycobacterial infection. In our case, three *M. smegmatis* infected quarters were not previously treated with intramammary antibiotics, thus in those cases it does not seem to be a predisposing factor.

Extended stress can negatively impact an animal’s health, primarily by suppressing the immune system and increasing disease susceptibility (56, 57). For example, heat stress in dairy cows leads to poor immunity (58), reduced production (58), and more intramammary infections (59). Similarly, noise and other disturbances from barn renovation can lower immune responses and lead to decreased milk yield, a higher somatic cell count (SCC), and poorer reproductive success (60, 61). We hypothesize that cows experience a more restless life in a research barn compared to a normal barn. On-going grazing experiments and the renovation of the barn may have influenced the cows’ disease susceptibility. However, more research is needed in this subject.

A Belgian study suggested possible cross-reactivity of infection with rapidly growing mycobacteria in paratuberculosis ELISA diagnostics (30). In their study, two cows having *M. smegmatis* mastitis displayed S/P values above the cut-off value in commercial ELISA detecting antibodies against MAP. However, it remained possible that the cows had concurrent MAP infections, despite the agent not being detected by other methods. In our study, similar results in MAP antibody diagnostics were observed. Moreover, we tested all 51 lactating cows. Four of the seven cows previously affected by *M. smegmatis* mastitis had been culled before sampling. But values above the cut-off were only observed in those other three, and MAP was not detected by qPCR in them. In Finland, MAP has never been detected in dairy herds, and the most recent MAP infection in beef cattle was detected in 2000. In addition, Finland has officially been free from bovine tuberculosis since 1995 (62, 63). Thus, concurrent MAP infection in our cases appears to be implausible. MAP control and monitoring programs have commonly been implemented in several countries. The key elements of the control programs include correct diagnostics. Our findings might indicate that false-positive serological results for MAP could be obtained in cattle with *M. smegmatis* mastitis.

In conclusion, NTM are uncommon bovine mastitis pathogens. We describe intramammary infections caused by genetically highly diverse *M. smegmatis* strains. In quarter level, chronic infections caused by persistent strains were observed. The WGS-based cgMLST approach proved to be a powerful tool for molecular epidemiological investigation of bovine NTM mastitis strains. Possible cross-reactivity due to infection with *M. smegmatis* was observed with a commonly used serological test for MAP, as cows with *M. smegmatis* mastitis tested positive in the MAP antibody ELISA test. Further investigations are recommended to determine the actual prevalence of *M. smegmatis* as a cause of mastitis in dairy cattle and its link to environmental factors like bedding materials.

## Data Availability

The datasets presented in this study can be found in online repositories. The names of the repository/repositories and accession number(s) can be found in the article/Supplementary material.
