# Supplementary material for: Cluster of Mycobacterium smegmatis mastitis cases in a dairy herd incorporating recycled manure solids bedding
Source: Front Vet Sci. 2026 Jan 26;12:1704276. doi: 10.3389/fvets.2025.1704276 (PMC12884540; doi:10.3389/fvets.2025.1704276)
Supplement: Supplementary file 1 [file Supplementary_file_1.pdf]

Genomes used for defining the scheme for the ad hoc cgMLST:

Seed Genome:

* NZ_CP054795.1 (17-DEC-2022), 6993871 bases, 6662 genes with CDS (Mycolicibacterium smegmatis strain FDAARGOS_679 chromosome)

Penetration Query Genomes (8):

* NZ_LN831039.1 (14-DEC-2022), 6983267 bases, 6647 genes with CDS (Mycolicibacterium smegmatis strain NCTC8159 chromosome 1, complete sequence)

* NZ_CP027541.1 (01-JAN-2023), 7119169 bases, 6769 genes with CDS (Mycolicibacterium smegmatis MKD8 chromosome, complete genome)

* NZ_CP082846.1 (17-DEC-2022), 6937705 bases, 6605 genes with CDS (Mycolicibacterium smegmatis strain FDAARGOS_1484 chromosome, complete genome)

* NZ_CP089215.1 (02-JAN-2023), 6891331 bases, 6543 genes with CDS (Mycolicibacterium smegmatis strain FDAARGOS_1618 chromosome, complete genome)

* NZ_CP080274.1 (14-MAR-2023), 6895172 bases, 6560 genes with CDS (Mycolicibacterium smegmatis strain Jucho chromosome, complete genome)

* NZ_CP080273.1 (14-MAR-2023), 7010278 bases, 6668 genes with CDS (Mycolicibacterium smegmatis strain Nishi chromosome, complete genome)

* NZ_CP080272.1 (14-MAR-2023), 7061747 bases, 6712 genes with CDS (Mycolicibacterium smegmatis strain Rabinowitchi chromosome, complete genome)

* NZ_CP089233.1 (02-JAN-2023), 7056740 bases, 6706 genes with CDS (Mycolicibacterium smegmatis strain FDAARGOS_1617 chromosome)

Exclude Sequences (1):

* NZ_CP054796.1 (17-DEC-2022), 1474 bases, 1 genes with CDS (Mycolicibacterium smegmatis strain FDAARGOS_679 plasmid unnamed)
